# The inflammatory response, a mixed blessing for muscle homeostasis and plasticity

**DOI:** 10.3389/fphys.2022.1032450

**Published:** 2022-11-23

**Authors:** Zineb Bouredji, Anteneh Argaw, Jérôme Frenette

**Affiliations:** ^1^ Centre Hospitalier Universitaire de Québec, Centre de Recherche du Centre Hospitalier de l’Université Laval (CRCHUQ-CHUL), Axe Neurosciences, Université Laval, Quebec City, QC, Canada; ^2^ Département de Réadaptation, Faculté de Médecine, Université Laval, Quebec City, QC, Canada

**Keywords:** muscle atrophy, sarcopenia, cachexia, inflammation, muscle-bone crosstalk

## Abstract

Skeletal muscle makes up almost half the body weight of heathy individuals and is involved in several vital functions, including breathing, thermogenesis, metabolism, and locomotion. Skeletal muscle exhibits enormous plasticity with its capacity to adapt to stimuli such as changes in mechanical loading, nutritional interventions, or environmental factors (oxidative stress, inflammation, and endocrine changes). Satellite cells and timely recruited inflammatory cells are key actors in muscle homeostasis, injury, and repair processes. Conversely, uncontrolled recruitment of inflammatory cells or chronic inflammatory processes leads to muscle atrophy, fibrosis and, ultimately, impairment of muscle function. Muscle atrophy and loss of function are reported to occur either in physiological situations such as aging, cast immobilization, and prolonged bed rest, as well as in many pathological situations, including cancers, muscular dystrophies, and several other chronic illnesses. In this review, we highlight recent discoveries with respect to the molecular mechanisms leading to muscle atrophy caused by modified mechanical loading, aging, and diseases. We also summarize current perspectives suggesting that the inflammatory process in muscle homeostasis and repair is a double-edged sword. Lastly, we review recent therapeutic approaches for treating muscle wasting disorders, with a focus on the RANK/RANKL/OPG pathway and its involvement in muscle inflammation, protection and regeneration processes.

## Introduction

Skeletal muscle is an essential organ of the human body. It is characterized by its great plasticity and capacity to adapt to numerous hormonal, nervous, and mechanical stimuli (Vittorio and Marcella, 2004). Skeletal muscle derives from a population of progenitor cells that originate in the somites of the paraxial mesoderm (Buckingham et al., 2003). Skeletal muscle plasticity is based on a balance between muscle hypertrophy, which results from an increase in organelles, cytoplasm, proteins and, consequently, an increase in the cross-section of muscle fibers, and muscle atrophy, which is associated with a decrease in cellular constituents and an increase in protein breakdown. Muscle breakdown involves several mechanisms, including the ubiquitin-proteasome system (UPS), which is regulated by E3 ubiquitin ligases, the autophagy-lysosome system (ALS), calpains, and caspases (Bonaldo and Sandri, 2013) (Yin et al., 2021). Many factors and situations cause protein degradation and/or a reduction in protein synthesis leading to muscle atrophy and wasting. Primary skeletal muscle atrophy is linked to genetic muscle disorders while secondary skeletal muscle atrophy is a consequence of physiological situations such as age-related sarcopenia, bed rest, and unloading, and pathological situations such as cancers, AIDS, osteoporosis, and heart failure (HF) (Yin et al., 2021). Muscle atrophy is also accelerated by the uncontrolled immune response observed in chronic diseases such as muscular dystrophies, including Duchenne muscular dystrophy (DMD), where the immune system promotes muscle cell death and fibrosis as the disease progresses (Tidball et al., 2018). However, the involvement of timely recruited inflammatory cells is a prerequisite for the activation and differentiation of satellite cells during the regeneration process (Tidball et al., 2018) (Dufresne et al., 2016). In this review, we highlight recent discoveries on the molecular mechanisms leading to muscle atrophy caused by modified mechanical loading, aging, and diseases. We also summarize current perspectives suggesting that the inflammatory process in muscle homeostasis and repair is a double-edged sword. Lastly, recent therapeutic approaches for treating muscle wasting disorders are reviewed, including pathways with potential impacts on skeletal muscle and bones.

## Overview of recent discoveries on the molecular mechanisms leading to muscle atrophy in healthy conditions

### Physical inactivity, unloading, and denervation-induced skeletal muscle atrophy

Prolonged bedrest and spaceflight-related unloading induce impaired muscle function, mainly due to the breakdown of myofibrillar proteins and the impairment of protein synthesis. In spaceflight or ground-based models of microgravity, antigravity muscles such as postural muscles atrophy due to a loss of myofibrillar proteins (Qaisar et al., 2020). Postural muscles, which are predominantly composed of slow-twitch type I fibers, are more affected than fast-twitch type II fibers following a period of unloading (Baldwin et al., 2013). Mechanistically, it has been suggested that selective atrophy is in part related to pre-transcriptional and transcriptional changes involving the downregulation of pre-mRNA and mRNA of myosin heavy chain (MHC) type I, actin, transcriptional enhancer factor-1 (TEF-1), and myogenin, which are mainly involved in protein synthesis (Baldwin et al., 2013). In addition to type I fiber atrophy, a switch in fiber phenotype from slow to fast is observed following hindlimb suspension (HS) and microgravity conditions. Epigenetic modifications of histones or non-coding RNAs would play a role in the changes in muscle phenotype (Pandorf et al., 2009) (Haddad et al., 2003). However, in humans, muscles with predominantly fast-twitch fibers appear to be as affected by microgravity as type I fiber muscles (Fitts et al., 2001). For example, the vastus lateralis muscle is predominantly composed of fast-twitch type II fibers, which are significantly more affected than slow-twitch type I fibers when humans are exposed to spaceflight for 11 days (Edgerton et al., 1995), suggesting that the preponderance of certain types of muscle fibers in a muscle may be more important than the individual fiber type in its responsiveness to unloading stimuli (Baldwin et al., 2013). In addition to muscle mass, a 20-day bedrest of human volunteers induced a significant decrease in quadriceps muscle thickness and cross-sectional area (CSA) and an increase in the expression of two ubiquitin ligase genes, Cbl-b and atrogin-1, which are involved in muscle proteolysis (Ogawa et al., 2006), suggesting that the mechanisms of muscle atrophy may differ depending on muscle location and function, fiber type, or the condition leading to the atrophy. In general, slow-twitch fibers are mostly affected by denervation, microgravity, and immobilization due to mechanical discharge while fast-twitch fibers are more affected by aging or disease conditions. As the oxidative metabolism of slow twitch fibers is mainly regulated by the transcription factor peroxisome proliferator-activated receptor-g coactivator-1 (PGC1a), the downstream calcineurin/nuclear factor of activated T cells (NFAT) may play a role in the protection of slow-twitch fibers from muscle atrophy in some pathological conditions (Wang and Pessin, 2013). Nonetheless, an imbalance between protein degradation and protein synthesis pathways remains at the origin of muscle atrophy.

**FIGURE 1:**
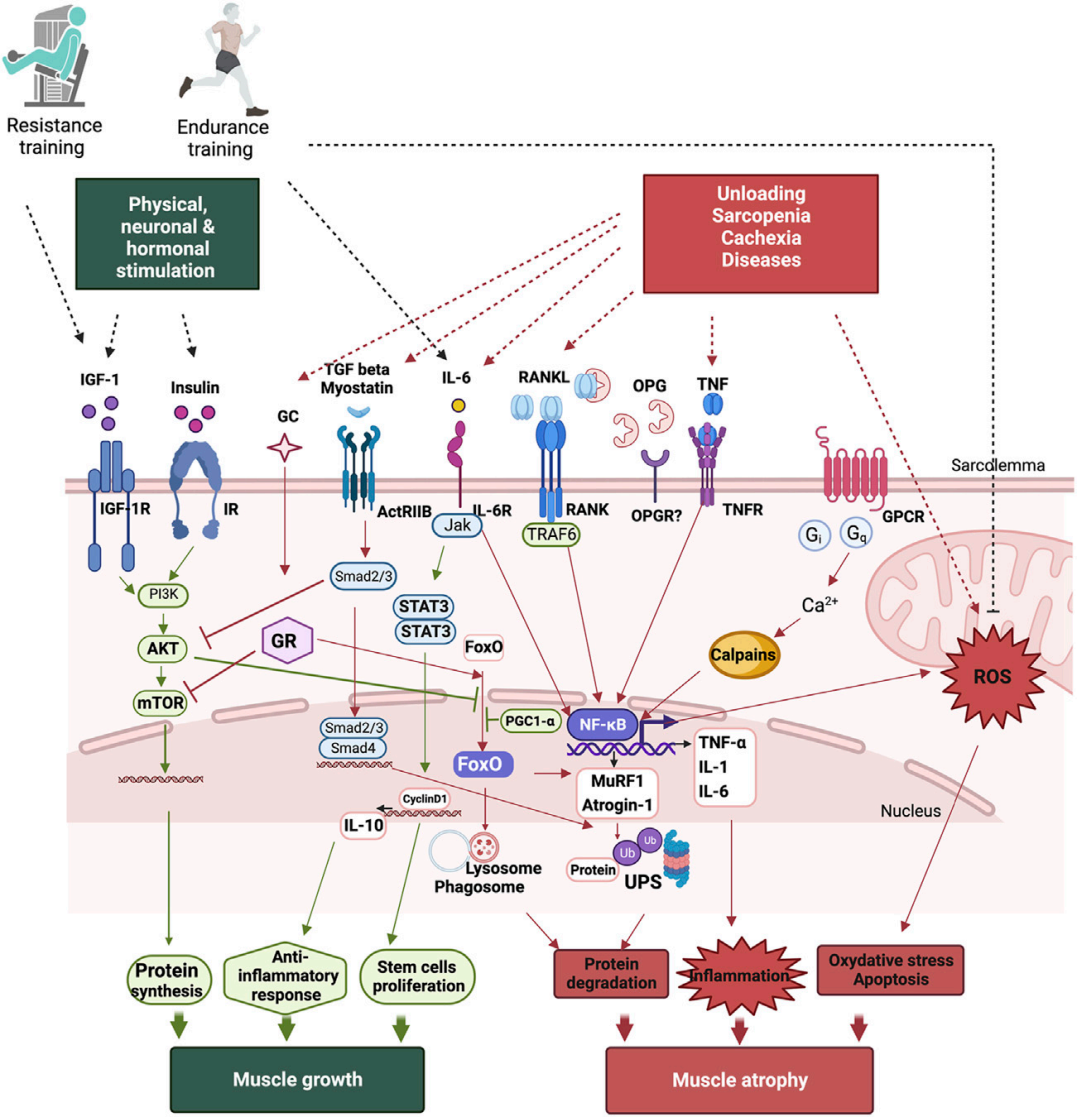
Signaling pathways leading to protein turnover in skeletal muscles. Muscle growth occurs following physical, neuronal, and hormonal stimulations. While a gain of muscle mass is mainly associated with resistance training, endurance training also improves muscle function by improving mitochondrial function, reducing oxidative stress, and modulating inflammation. Following unloading, age-related muscle wasting, sarcopenia, or diseases such as cachexia induces muscle atrophy. Muscle atrophy occurs as result of an increase in protein degradation, inflammation, oxidative stress, and apoptosis. The main pathway involved in protein synthesis is the PI3K/Akt/mTOR pathway, which is activated both by growth hormone (GH) and insulin-like growth factor IGF-1. Protein breakdown involves the activation of the ubiquitin-proteasome system (UPS) and the autophagy-lysosome system (ALS), which are mainly regulated by FoxOs and NF-κB, leading to muscle atrophy. NF-κB also inceases pro-inflammatory cytokine production and ROS, accentuating muscle loss. TGF beta, myostatin, RANKL, TNF-α, and IL-6 induce NF-κB activation. However, IL-6 can act as both a pro- and an anti-inflammatory cytokine as it induces anti-inflammatory cytokine IL-10 production and is mainly involved in stem cell proliferation leading to muscle growth.

The insulin/IGF-1/Akt-mTOR pathway is the main pathway regulating muscle growth (Figure 1). When insulin and IGF-1 bind to their respective receptors, they initiate a phosphorylation cascade that leads to the activation of Akt, also known as protein kinase B. Akt enhances protein synthesis *via* the activation of the mammalian target of rapamycin (mTOR) pathway and the inhibition of glycogen synthase kinase β (GSK3β). Additionally, Akt blocks forkhead box Os (FoxOs), a family of transcription factors, preventing protein degradation (Sartori et al., 2021) (Schiaffino and Mammucari, 2011). It has been reported that the IGF-1/PI3K/Akt signaling pathway reduces denervation-, unloading-, and joint immobilization-induced muscle atrophy, and that the injection or overexpression of IGF-1 counteracts denervation- and age-related muscle atrophy (Timmer et al., 2018). On the other hand, several cellular systems are responsible for ensuring the turnover of muscle proteins and are finely regulated by complex regulatory mechanisms (Figure 1). UPS remains the primary system for protein breakdown in all tissues (Rock et al., 1994). The labeling of proteins by a ubiquitin molecule leads to their recognition and degradation by the 26S proteasome. Ubiquitination is ensured by three enzymes (E-1, E-2, and E-3). E3 is the key enzyme as it recognizes the substrate and catalyzes the insertion of ubiquitin (Lecker et al., 2006). The cellular level of E3 ligases determines protein degradation. Two E3 ligases, atrogin-1 and MuRF-1, are specific constituents of muscle, and their expression increases considerably in catabolic states (Lecker et al., 2006). Accordingly, FoxOs are key players in the regulation of muscle atrophy. FoxO 1, 3, and 4 regulate the induction of atrophy-related genes by controlling the ALS and UPS (atrogin-1 and MuRF1) (Brocca et al., 2017) (Bonaldo and Sandri, 2013). Akt phosphorylates FoxOs, inducing their inhibition and exportation from the nucleus thereby preventing muscle atrophy (Calnan and Brunet, 2008). The expression of FoxO-dependent atrophy-related genes varies in different muscles under different conditions. FoxO-1, -3, or -4 deletion in mice significantly prevents the loss of muscle mass following unloading (Brocca et al., 2017) but only partially spares skeletal muscle following denervation (Milan et al., 2015), highlighting the divergence in the mechanisms of the atrophy program in each catabolic condition. Calpains are also involved in inactivity-induced muscle atrophy since their inhibition attenuates muscle atrophy in rodents (Talbert et al., 2013) (Hyatt and Powers, 2020). Transgenic overexpression of calpastatin, the endogenous calpain inhibitor, protects skeletal muscle against atrophy suggesting their involvement in muscle wasting (Salazar et al., 2010) (Tidball and Spencer, 2002). Disruption in cytosolic Ca^2+^ homeostasis plays a role in initiating calpain activation following physical inactivity (Ingalls et al., 2001), and reactive oxygen species (ROS) generation exacerbates the rise in cytosolic Ca^2+^ leading to further calpain activation (Hyatt and Powers, 2020). More recently, Hayden and colleagues provided evidence that calpains play an important function in inactivity-induced mitochondrial dysfunction and oxidative stress in skeletal muscle fibers (Hyatt et al., 2021). They showed that the overexpression of calpastatin inhibits calpain activation thereby reducing mechanical ventilation-induced oxidative stress and mitochondrial dysfunction, fiber atrophy and contractile dysfunction (Hyatt et al., 2021).

In addition to the dysregulation of protein turnover, muscle stem cell proliferation and differentiation also decrease, which may contribute to atrophy under unloading conditions. Muscle atrophy leads to a significant decrease in the number of stem cells, with alterations in their capacity to proliferate and differentiate, accentuating muscle wasting in a mouse model of HS (Mitchell and Pavlath, 2004). Furthermore, the Notch signaling pathway, which tightly regulates stem cell proliferation and quiescence but not differentiated myofibers, is also involved in the alteration of regeneration and muscle atrophy in aging and muscular diseases (Gioftsidi et al., 2022). A recent study has shown that the inhibition of Notch signaling attenuates atrophy and fibrosis and reduces fibroblast levels in denervated gastrocnemius muscles (Feng et al., 2019). The mechanisms of muscle atrophy are thus complex and involve a panoply of signaling pathways. The specificities of the molecular mechanisms are still being investigated.

### Age-related skeletal muscle atrophy: Sarcopenia

Sarcopenia is characterized by a progressive age-related loss in muscle mass and strength limiting motor function and a decrease in quality of life. Muscle atrophy and muscle cell death are the primary cause of the loss in muscle mass due to many molecular and cellular changes, including changes in mitochondria function, oxidative stress, hormonal signaling, and inflammatory cytokine secretion (Roubenoff, 2000). At the cellular level, mitochondrial function deteriorates in sarcopenia. Mitochondrial dysfunction involves various processes, including the production of ROS, mitochondrial biogenesis and turnover, Ca^2+^ dynamics, energy sensing, and apoptosis (Gonzalez-Freire et al., 2015). Recently, Grevendonk *et al.* (2021) examined mitochondrial function in young and elderly patients and determined whether physical activity interferes with mitochondrial dysfunction. The elderly patients exhibited a lower mitochondrial capacity compared to young adults with a similar mitochondrial content and who had performed comparable physical exercise. The muscle strength, aerobic capacity, exercise efficiency, insulin sensitivity, and gait stability were lower in elderly adults with higher body fat. However, an increase in physical activity partially reversed these observations (Grevendonk et al., 2021). In addition to mitochondrial dysfunctions, many studies have reported that age-related decreases in nicotinamide adenine dinucleotide (NAD+) accentuate muscle wasting (Yaku et al., 2018) (Castro-Portuguez and Sutphin, 2020). NAD+ is an important co-enzyme that regulates the energy balance, oxidative stress and protein posttranslational modifications. Aging disrupts both NAD + synthesis and degradation. Camacho-Pereira and colleagues shows that the decrease in NAD+ in aging is related to an increased level of CD38; the main enzyme involved in NAD + degradation (Camacho-Pereira et al., 2016). In addition, they showed that CD38 is also involved in mitochondrial dysfunction through a mechanism involving SIRT3; a regulator of mitochondrial homeostasis. In murine, the deletion of Nampt, an essential enzyme in the NAD + salvage pathway, induces an 80% decrease in intramuscular NAD + content which is accompanied by muscle degeneration and progressive loss of muscle strength and function. In contrast, the lifelong overexpression of Nampt preserved muscle NAD + levels and exercise capacity in aged mice, highlighting the important role played by NAD+ in muscle homeostasis (Frederick et al., 2016). In other tissues, PARP-mediated NAD^+^ depletion and loss of SIRT1 activity have been associated with an increase in oxidative nuclear damage and oxidative stress in both rodents and human (Massudi et al., 2012). Autophagy is another cellular mechanism that is modified with aging and that results in muscle atrophy (Wohlgemuth et al., 2010). Autophagy ensures that cellular components are delivered into lysosomes to be degraded and recycled, ensuring cellular homeostasis (Barbosa et al., 2019). However, autophagic activity decreases with age, increasing the production of ROS and oxidative stress causing sarcopenia (Barbosa et al., 2019) and reducing the ability of satellite cells to participate in muscle regeneration during aging (see review Chen et al., 2022). Activating transcription factor 4 (ATF4) also participates in age-related skeletal muscle atrophy (Ebert et al., 2015). ATF4 interacts with both C/EBPβ and the ATF–C/EBP composite site to activate *Gadd45a,* a gene involved in stress-related growth arrest and muscle atrophy (Ebert et al., 2020). In terms of muscle phenotype, type II muscle fibers are mostly atrophied in aging, also causing a marked conversion from the fast-to the slow-twitch phenotype (Dao et al., 2020) (Nilwik et al., 2013) (Ciciliot et al., 2013). The decline in satellite cell content may in part explain age-related type II muscle fiber atrophy (Verdijk et al., 2014). Thus, age-related muscle atrophy is very complex and multiple pathways converge toward muscle atrophy along with a reduced muscle regeneration capacity.

The age-related impairment of the motor unit (MU) is also responsible for the loss of muscle mass and function due to motoneuron loss, dysfunction in the process of compensatory reinnervation, and alterations at the neuromuscular junctions (NMJ). Larsson and colleagues (2019) recently presented a detailed review demonstrating the age-related changes in the MU in animals and humans (Larsson et al., 2019). It has been reported that the number of MUs is reduced by more than 40% after the age of 71, causing progressive muscle fiber denervation (Piasecki et al., 2016). A reinnervation mechanism is often set up at an advanced age favoring an inhomogeneity in the distribution of MUs (Larsson et al., 2019). However, this compensatory system is often insufficient to counteract the loss of motoneurons leading to the death of muscle fibers. In addition, the neuronal conduction and the quality of transmission are impaired in aging motor neurons (Larsson et al., 2019). Structural and molecular changes such as the reduction in the stability of transmission, the presence of fragmentation and the alteration of primary signaling molecules like the nicotinic acetylcholine receptor and agrin have been reported in aging NMJ (Rudolf et al., 2014) (Hepple and Rice, 2016). Skeletal muscle capillarization is also reduced during aging and the distance between satellite cells and capillaries increases potentially impacting the capacity for muscle regeneration in humans (Joanisse et al., 2017) (Larsson et al., 2019). Muscle capillarization is accompanied by a decrease in mitochondrial enzyme function, highlighting the limitations in oxidative capacity (Coggan et al., 1992). Thus, aging-related morphological changes occurring at the MU and capillarization may contribute directly or indirectly to molecular mechanisms responsible for muscle atrophy.

Changes in anabolic hormone secretion also play an important and easily observed role in sarcopenia. Growth hormone (GH), testosterone, and insulin-like growth factor (IGF-1) secretion decline gradually with age (Bian et al., 2020). GH binds directly to its receptor, activating the JAK2/STAT pathway and myocyte proliferation (Skorupska, 2018) (Velloso, 2008) and maintains muscle mass by promoting IGF-1 secretion by the liver, a key anabolic hormone (Chikani and Ho, 2014) (Widdowson and Gibney, 2008). In addition to the decrease in anabolic hormone secretion, anabolic resistance slowly develops as the anabolic response to stimulation is attenuated with aging, reducing the sensitivity to IGF-1 stimuli and the Akt-mTOR cascade thus limiting the capacity of muscles to grow (Dao et al., 2020) (Funai et al., 2006). Moreover, IGF-1 modulates mitochondrial functions through the activation of the Akt pathway and the inhibition of FoxO proteins (Poudel et al., 2020). The local expression of IGF-1 protects against the age-related decline in muscle force and mass. Transgenic mice revealed that the local expression of IGF acts by increasing the ALS and the expression of PGC1-α, which modulates mitochondrial function and ROS detoxification in aged mice (Ascenzi et al., 2019). In addition, the levels of myostatin, a member of the TGF-β superfamily and a negative regulator of skeletal muscle mass, increase in aging men and women (Yarasheski et al., 2002) and may potentially be an important regulator of muscle wasting. Several alterations that occur with aging disturb the protein turnover process and lead to protein breakdown and muscle atrophy. All these changes in the hormonal profile mainly influence muscle growth and lead to sarcopenia during aging.

Sarcopenia is characterized by chronic low-level inflammation that develops with advanced age, which is also called inflammaging. This inflammation may contribute to muscle loss associated with aging. Pro-inflammatory cytokines, including members of the TNF family, as well as downstream transcription factor NF-κB, are significant regulators of muscle atrophy (Thoma and Lightfoot, 2018). The NF-κB signaling pathway increases the expression of several proteins of the UPS involved in the degradation of specific muscle contractile proteins and can also interfere with myogenic differentiation (Li et al., 2008) (Thoma and Lightfoot, 2018) (Cai et al., 2004). Furthermore, Pasco *et al.* (2020) recently summarized the mechanisms of muscle atrophy involving the weak inducer of TNF-like apoptosis (TWEAK) and its associated receptor, fibroblast growth factor 14 (Fn14). The TWEAK/Fn14 signaling pathway maintains chronic inflammation and induces the secretion of profibrotic cytokines (Zhang et al., 2021). Fn14 levels increase in aged mice while the genetic deletion of Fn14 increases specific muscle protein levels and reduces age-related fiber atrophy, most likely by decreasing the DNA-binding activity of NF-κB (Tajrishi et al., 2014). Additionally, TNF-α enhances ROS generation in muscle cells, causing muscle wasting, while antioxidant treatments inhibit TNF-α-induced atrophy (Xu et al., 2018). NF-κB and chronic inflammatory pathways thus play significant roles in skeletal muscle atrophy under physiological and pathological conditions. Acute and well-controlled inflammation remains essential for muscle repair, regeneration, and growth. Its dual function is discussed in the following section.

## Overview of the inflammatory process in skeletal muscle: A double-edged sword

### The role of inflammation in muscle repair

The presence of satellite cells and inflammatory cells plays a major role in the skeletal muscle regenerative process (Tidball, 2017). The participation of sentinel inflammatory cells such as mast cells are important for the initiation of the inflammatory process following an injury (Perandini et al., 2018) (Bentzinger et al., 2013) (Gaudenzio et al., 2016). Mast cells secrete inflammatory mediators and lead to a well-orchestrated sequential activation and recruitment of neutrophils that play an important role in fighting pathogens (Kawanishi et al., 2016). However, in cases of severe injuries, the release of ROS and the secretion of proteolytic enzymes resulting from prolonged activation of neutrophils may cause collateral damage (Duchesne et al., 2017) (Dumont et al., 2008). The pro-inflammatory environment is crucial for the arrival of monocytes that differentiate into pro-inflammatory M1 macrophages in injured skeletal muscle. M1 macrophages secrete pro-inflammatory cytokines. Including TNF-α and IL-1β, and play a key role by ensuring the phagocytosis of cell debris and apoptotic neutrophils, the activation and proliferation of satellite cells, and the recruitment of other immune actors (Locati et al., 2013) (Mosser and Edwards, 2008) (Otis et al., 2014). Pro-inflammatory M1 macrophages switch to the anti-inflammatory M2 macrophage phenotype, which promotes the differentiation of satellite cells and the formation of mature myofibers. M2 macrophages initiate muscle repair by secreting anti-inflammatory cytokines (IL-4, IL-10) and growth factors (TGF-β, IGF-1, and HGF) (Arnold et al., 2007) (Chazaud et al., 2003) (Deng et al., 2012) (Oishi and Manabe, 2018). They are important for promoting muscle regeneration and repair following acute muscle injuries (Chazaud et al., 2003) (Arnold et al., 2007) (Chazaud, 2020) (Bouredji et al., 2021). In addition to cytokines, lipid mediators generated *via* cyclooxygenase-2 (COX-2) also play a prominent role by orchestrating the inflammatory process and contributing to the macrophage switch and inflammation resolution (Duchesne et al., 2017). The depletion of immune cells or COX-2 inhibition impairs muscle regeneration and induces fibrosis during acute inflammatory processes, highlighting the predominant role of inflammation in muscle healing. This puts into question the relevance of using anti-inflammatory treatments for acute muscle injuries (Toumi et al., 2006) (Summan et al., 2006) (Duchesne et al., 2017) (Bondesen et al., 2004).

### Cytokines and their impact on skeletal muscle

Discoveries over the past 20 years have significantly improved our understanding of myokines, i.e., interleukins, growth factors, and the numerous peptides secreted by muscles (Pedersen, 2013). Interleukin 6 (IL-6) is one of the most thoroughly studied myokines. Considered, in part, as a pro-inflammatory cytokine, human muscle contractions release a significant amount of IL-6, depending on the duration and intensity of the physical activity (Pedersen, 2013). IL-6 secreted during exercise has anabolic properties and participates in the regulation of glucose and lipid metabolism (Ellingsgaard et al., 2019). IL-6 is an essential regulator of satellite cell-mediated muscle growth while the deletion of the IL-6 gene attenuates muscle hypertrophy and reduces the proliferation of satellite cells *via* STAT3 as well as the expression of its target, cyclin D1 (Serrano et al., 2008). Chowdhury *et al.* (2020) recently showed that the high levels of IL-6 found in circulation during physical exercise mainly originates from muscle. Furthermore, muscle-released IL-6 increases RANKL secretion by osteoblasts, promoting osteoclast differentiation and osteocalcin release. In turn, circulating osteocalcin potentiates IL-6 released by muscle cells, increasing nutrient uptake and exercise capacity in rodents (Chowdhury et al., 2020). IL-6 is also an anti-inflammatory cytokine and promotes the expression of another anti-inflammatory myokine, IL-10, which inhibits the production of proinflammatory cytokines, including IL-1 and TNF-α, during regular exercise (Petersen and Pedersen, 2005), favoring the switch of macrophages to anti-inflammatory M2 macrophages during muscle repair and regeneration (Deng et al., 2012). However, IL-6 is also associated with numerous skeletal muscle alterations such as atrophy and muscle wasting. The IL-6/JAK/STAT3 signaling pathway is strongly activated during denervated skeletal muscle atrophy. IL-6 enhances C2C12 myotube atrophy while pharmacological blocking of IL-6 reduces the expression of atrophic and autophagy-related genes (Huang et al., 2020) (Madaro et al., 2018). Another study has recently shown that IL-6 is upregulated in the skeletal muscles of limb-immobilized mice and that a systemic IL-6 deficiency protects against immobilization-induced muscle atrophy (Hirata et al., 2022). IL-15 is also abundantly expressed and secreted by skeletal muscle in response to exercise (Huang et al., 2015) (Yang H et al., 2013) and protects muscle against sepsis-induced muscle wasting (Kim et al., 2012). *In vitro*, IL-15 overexpression induces myotube hypertrophy similar to the overexpression of IGF-1 (Quinn et al., 2002) while the co-incubation of IL-15 and TNF-α reduces the detrimental effects of TNF-α on myotube development (O’Leary et al., 2017), highlighting the potential of IL-15 to prevent muscle wasting. However, the hypertrophic effects of IL-15 seem to be effective in pathological situations, and IL-15 is not an anabolic factor in homeostasis and steady state conditions (Nadeau and Aguer, 2019) where anabolic effects on the muscle mass of mice overexpressing IL-15 are not observed (Pistilli and Quinn, 2013). IL-15 is also involved in bone development, and mice lacking IL-15 receptor alpha (IL-15 RA) exhibit a decrease in bone mineralization (Loro et al., 2017). Myostatin, another myokine and a member of the TGF-β superfamily, negatively regulates muscle growth. The myostatin/TGF-β/activin pathway *via* activin receptor type II (ActRIIB) induces the activation of SMAD2/3 causing the inhibition of Akt, protein synthesis, and cell differentiation (Morissette et al., 2009) (Trendelenburg et al., 2009). In addition, myostatin deletion increases both muscle mass and bone density (DiGirolamo et al., 2015). More recently, ActRIIB signaling has been shown to directly and negatively regulate bone mass in osteoblasts, suggesting that myostatin neutralization would benefit bones (Goh et al., 2017). Although the receptor activator of nuclear factor kappa B (RANK), its ligand (RANKL), and osteoprotegerin (OPG), a decoy soluble receptor of RANKL, constitute the primary pathway regulating bone homeostasis, we have shown over the last 10 years that this triad plays significant roles in skeletal muscle disease and repair. RANKL, through the activation of the NF-κB pathway, is involved in the activation of inflammatory-atrophic pathways in skeletal muscle (Hamoudi et al., 2019). Muscle cells in culture also produce and secrete OPG (Dufresne et al., 2015), and its deletion in mice causes muscle atrophy (Hamoudi et al., 2020). Pharmaceutical treatments with recombinant OPG markedly improve muscle integrity and function in dystrophic mice (Dufresne et al., 2015) (Dufresne et al., 2018) and in a mouse model of acute muscle injury (Bouredji et al., 2021). Our recent review discusses the involvement of the RANK/RANKL/OPG triad in skeletal and cardiac muscles in greater detail (Marcadet et al., 2022).

### Disease-related skeletal muscle atrophy

#### Cancer cachexia

While sarcopenia is characterized by a loss of muscle mass and strength with aging, cachexia is a condition characterized by a continuous decrease in skeletal muscle mass, involuntary weight loss, and the deterioration of an individual’s nutritional status, and is mainly observed in chronic diseases such as cancer (Anker et al., 2019) (Dolly et al., 2020). Perturbations of cellular and inflammatory processes, including the activation of inflammation and proteolytic mechanisms, UPS and the ALS are at the origin of cancer-associated cachexia (Baracos et al., 2018). The central mechanism behind cancer-associated cachexia is inflammation (Cortiula et al., 2022) involving the overproduction of inflammatory cytokines, especially IL-6, TNF-α, and IL-1β. The simultaneous secretion of IL-6 and TNF-α induces muscle weakness as well as the activation of pro-inflammatory pathways, including the NF-kB pathway (Webster et al., 2020). Cachexia is associated with high levels of circulating cytokines, and preclinical studies have shown that there is an increase in UPS and a decrease in protein synthesis due to the downregulation of Akt and FoxO3 phosphorylation (Webster et al., 2020). A recent clinical study involving gastric cancer patients with cachexia has shown that the patients exhibit a significant decrease in the CSA of skeletal muscle associated with a significant increase in UPS and ALS (Zhang et al., 2020). The current hypothesis regarding cancer-associated cachexia suggests that mitochondrial dysfunction combined with lipid droplet accumulation and oxidative stress increases proteolysis and the loss of muscle mass through UPS and ALS (Dolly et al., 2020). The RANKL/RANK interaction has recently been associated with muscle wasting and cachexia in a mouse model of non-metastatic cancer. Fabrizio *et al.* (2022) have shown that elevated levels of circulating RANKL are sufficient to cause skeletal muscle atrophy and bone resorption whereas anti-RANKL treatments improve muscle mass and function in cancer-associated cachexia (Pin et al., 2022). The NF-kB pathway is also involved in impairing satellite cell differentiation in cancer patients and animal models by inducing PAX7 dysregulation, impairing muscle regeneration, and promoting muscle wasting (He et al., 2013). Moreover, therapeutic approaches, including chemotherapy or radiotherapy, can potentially induce muscle atrophy and dysfunction. Chemotherapeutic compounds induce muscle wasting in mice through the activation of the NF-kB pathway (Damrauer et al., 2018) (Coletti, 2018). Some 218 proteins are downregulated in C26-tumor bearing mice and chemotherapy-treated mice. A pathway analysis has shown showed that these two conditions both lead to the dysregulation of mitochondrial functions, the Krebs cycle, and fatty acid metabolism as well as Ca^2+^ dysfunctions and that both are associated with muscle loss (Barreto et al., 2016). Furthermore, cancer cells and the administration of cytotoxic chemotherapeutics induce a rapid inflammatory response, leading to the dysregulation of the neuroendocrine system, more precisely the hypothalamic-GH-IGF-1 axis. The inhibition of this axis contributes to skeletal muscle atrophy and cachexia (Martín et al., 2021). In addition, a rapid inflammatory response induced by cytotoxic chemotherapy treatment leads to endogenous glucocorticoid (GC) secretion, which increases in the UPS-, lysosome- and autophagy-genes, resulting in muscle loss (Braun et al., 2014). Conversely, the muscle-specific deletion of the GC receptor blocks treatment-induced muscle atrophy, highlighting the involvement of GCs in cachexia (Braun et al., 2014) (Braun et al., 2013). However, the mechanisms of chemotherapy-induced cachectic myopathy may be specific to each chemical agent and has recently been reviewed by Campelj *et al.* (2021)(Campelj et al., 2021). Overall, cancer-related cachexia is a consequence of many disorders caused by cancerous cells and the side effects of therapeutic approaches, including the reaction of the body against the tumor, the inflammatory reaction, the impairment of metabolism, and the loss in appetite.

#### Heart failure-related skeletal muscle dysfunction/atrophy

Muscle wasting often occurs concomitant with chronic diseases. Patients suffering from chronic HF present marked muscle atrophy, with a reduction in exercise capacity and muscle strength (Fülster et al., 2013). Similarly, 47% of young patients with HF experience muscle wasting (Hajahmadi et al., 2017). Metabolic and molecular changes, in particular protein breakdown and an increase in the production of ROS, may explain the muscle dysfunction/exercise intolerance following HF (Springer et al., 2017) (Nambu et al., 2021) (Wood et al., 2021). MuRF-1 is increased in the skeletal muscle of HF patients and physical activity attenuates its expression (Gielen et al., 2012). The inhibition of MuRF1 has a protective effect on muscle atrophy in mice with HF (Adams et al., 2019). In addition, the expression of IGF-1 in skeletal muscle is reduced (Hambrecht et al., 2002) and, consistent with these findings, the phospho-Akt/Akt levels and phospho-mTOR/mTOR ratios are diminished in patients with HF compared with healthy controls of a similar age, muscle mass, and physical activity levels. Further investigations are clearly needed to better understand the molecular mechanisms regulating HF-induced muscle wasting and exercise intolerance.

#### Muscle wasting in muscular dystrophies

A common characteristic of genetic muscle diseases, including congenital muscular dystrophy and mitochondrial and genetic myopathies, is a major impairment of muscle integrity and the loss of Ca^2+^ homeostasis, which in turn leads to a loss of muscle function (Yin et al., 2021). In myotonic dystrophy type 1 (DM1), a genetic disease that causes myotonia, muscle atrophy, and insulin resistance, insulin receptor type 1 is expressed at lower levels in skeletal muscle, which leads to a decrease in mTOR signaling and an increase in UPS protein expression, giving rise to muscle atrophy (Renna et al., 2019). Congenital muscular dystrophy caused by genetic changes in the LAMA2 gene is mainly characterized by severe muscle wasting. Preclinical studies have shown that the increase in protein degradation is the result of an increase in UPS protein expression and a reduction in Akt phosphorylation and protein synthesis (Carmignac et al., 2011) (Gawlik et al., 2019). DMD is one of the most common forms of muscular dystrophy. Although the alterations in muscle integrity and function lead to progressive muscle atrophy in DMD, certain muscles may grow as a result of an accumulation of fat and fibrosis and an increase in myofiber size called compensatory hypertrophy (Kornegay et al., 2012). In the mdx mouse model of DMD, an initial phase of muscle hypertrophy is followed by atrophy associated with a decline in regenerative potential and changes in the activation of the mTOR signaling pathway (Mouisel et al., 2010). In both animals and humans, dystrophic muscles are not uniformly affected, with some being atrophied others hypertrophied, adding a greater challenge for therapeutic healthcare (Kornegay et al., 2012). Additionally, membrane fragility due to the absence of dystrophin leads to chronic inflammation, which aggravates the pathology (Petrof et al., 1993) (Wehling et al., 2001). Patients affected by DMD exhibit an increased expression of inflammatory cells (Abdel-Salam et al., 2009) while chronic inflammation alters the macrophage switch. Macrophages express high levels of transforming growth factor β1 (TGF-β1), which disrupts muscle regeneration and increases connective tissue deposition (Lemos et al., 2015) (Tidball, 2017). Neutrophils and mastocytes also play a harmful role in dystrophic muscles, and their depletion reduces necrosis (Hodgetts et al., 2006) (Radley and Grounds, 2006), whereas regulatory T cells modulate the progression of muscular dystrophy by suppressing the pro-inflammation environment in dystrophic muscles (Villalta et al., 2014). A recent review summarizes the harmful role of inflammatory cells in muscular dystrophies and describes an immune response involving neutrophils, macrophages, helper T-lymphocytes, and cytotoxic T-lymphocytes (Tidball et al., 2018).

The standard of care for patients with muscular dystrophies remains the chronic use of GCs. Although GCs are the only therapeutic agents with proven benefits for quality of life, mobility, and life expectancy, they are associated with several adverse effects, including potential muscle atrophy (Goemans and Buyse, 2014). GCs induce selective fast-twitch glycolytic fiber atrophy typified by a reduction in CSA and protein content. GCs modulate IGF-1 and myostatin signaling and induce the increase in protein breakdown through the UPS and lysosomal systems involving Murf-1, Atrogin-1, and FoxO (Schakman et al., 2013). Furthermore, GCs interfere with protein synthesis by inhibiting the mTOR/S6 kinase 1 pathway (Schakman et al., 2013) (Braun and Marks, 2015). Other mechanisms that contribute to GC-induced muscle atrophy involve the downregulation of the transcription factors myoD and myogenin, and the increase in insulin resistance and Ca^2+^ levels through activated store-operated Ca^2+^channels (SOCs) (Hasselgren et al., 2010). A recent study has further highlighted the adverse effects of GCs on bone integrity (Box et al., 2022). GC-related bone demineralization is mainly caused by a reduction in bone formation combined with an increase in bone resorption (Box et al., 2022). GCs inhibit osteoblastogenesis and the activity of osteoblasts and osteocytes (Weinstein et al., 1998). They also inhibit OPG secretion by transrepressing the OPG gene, which increases the RANKL/OPG ratio, and by promoting osteoclast proliferation and bone resorption (Kondo et al., 2008) (Jia et al., 2006). Prolonged and chronic use of GCs in DMD is thus potentially associated with muscle atrophy and bone demineralization.

#### Bone disease-related skeletal muscle dysfunction/atrophy

Osteoporosis is characterized by a reduction in bone mass associated with microarchitectural deterioration of bone tissue due to an imbalance between bone resorption and bone formation, which occur simultaneously with sarcopenia (Reginster et al., 2016). The occurrence of osteoporosis was, for a long time, considered a consequence of sarcopenia associated with an age-related reduction in mobility. However, major advances point to bidirectional molecular communication between bone and muscle that share common signaling pathways, suggesting a concomitant wasting of bone and muscle (Reginster et al., 2016). Terracciano *et al.* (2013) have shown that patients suffering from osteoporosis present a preferential and diffuse type II fiber atrophy that is proportional to the degree of bone loss and possibly linked to a reduction in Akt expression (Terracciano et al., 2013). Our laboratory has investigated bone-muscle bi-directional communication, with a focus on RANK/RANKL/OPG, the main pathway regulating bone homeostasis. The dysregulation of this pathway is directly associated with the onset of osteoporosis (Ono et al., 2020). Preclinical studies in mice have shown that RANKL/RANK interactions are involved in muscle wasting, atrophy, and dysfunction (Box et al., 2022) (Hamoudi et al., 2020) (Dufresne et al., 2018) (Bonnet et al., 2019). Our work highlights the involvement of the RANK/RANKL/OPG pathway in mitigating DMD. Moreover, OPG knockout mice exhibit selective atrophy of fast-twitch-type IIb myofibers, the most powerful muscle fibers, which may occur through the activation of the NF-kB pathway (Hamoudi et al., 2020). *In vitro*, RANKL stimulation of myotubes in culture increases the expression of NF-kB, atrogin-1, and MuRF-1 (Hamoudi et al., 2020). Furthermore, mice overexpressing RANKL exhibit decreased muscle mass, force, and glucose uptake associated with an upregulation of inflammatory genes (Bonnet et al., 2019). In a mouse model of sarcopenia, RANKL neutralization increases muscle volume and force and normalizes insulin signaling and inflammatory genes in skeletal muscle (Bonnet et al., 2019). Our recent review summarizes the current knowledge on the involvement of RANK/RANKL/OPG signaling in cardiac, skeletal, and smooth muscles in health and disease (Marcadet et al., 2022). The molecular mechanisms concertedly regulating muscle wasting and bone diseases are now being intensively investigated and important findings will soon emerge.

## Overview of the therapeutic approaches for treating muscle wasting

### Exercise, electrical stimulation and nutrition

Exercise is the most effective treatment for counteracting muscle wasting and atrophy (Glass and Roubenoff, 2010). Resistance training induces muscle hypertrophy in part by increasing the expression of IGF-1 (Glass and Roubenoff, 2010). The overexpression of IGF-1 or its addition to cultures of myotubes increases myofiber size. IGF-1 induces an increase in muscle mass by promoting protein synthesis through PI3K-Akt-mTOR signaling and inhibiting FoxO transcription factors involved in myofibril degradation. The expression of hypertrophic factor PGC1α increases with exercise training, reducing the FoxO3-dependent loss in muscle mass (Sandri et al., 2006). While resistance exercise induces skeletal muscle hypertrophy by promoting growth signaling pathways, endurance training is also beneficial for improving the capillary and mitochondrial networks and promoting the switch in muscle fiber phenotype towards more oxidative fiber isoforms thereby improving muscle physiology in HF (Nijholt et al., 2022). More recently, swimming has been shown to attenuate tumor growth and cancer-related muscle atrophy by downregulating the expression of proinflammatory proteins including NF-κB, p-NF-κB, TNF-α, IL-1β, and IL-6 (Li et al., 2021). The anti-inflammatory effects of exercise are increasingly being investigated and are of major interest for the treatment of chronic inflammation and muscle atrophy (Suzuki, 2019) (He and Ye, 2020). However, resistance training cannot be prescribed for all patients, in particular to those who are bed-ridden with acute disease or frail elderly sarcopenic individuals. In addition, exercise must be performed on a regular, long-term basis to maintain its effectiveness. Additionally, the transcutaneous neuromuscular electrical stimulation (NMES), a biophysical agent, may be beneficial to slow down muscle atrophy in deconditioning patients. For example, NMES was found to have benefits in terms of improved muscle mass and function and health status for patients with limited physical capacity (Gerovasili et al., 2009). In bed-ridden patients suffering from a severe COPD, NMES associated with an active limb mobilization significantly improve muscle strength and life quality (Zanotti et al., 2003). The same results were obtained in a subsequent study (Vivodtzev et al., 2006) confirming the benefice of NMES when combined with rehabilitation program. In patient with advanced chronic heart failure, the use of a low-frequency NMES helps to increase exercise capacity and to counteract skeletal muscle deterioration (Nuhr et al., 2004). Furthermore, the NMES of lower extremities preserves the muscle mass in critically ill patients (Gerovasili et al., 2009). Although NMES seems to improve muscle function particularly in patients with limited physical capacity, the methodological quality is generally poor and the heterogeneity in the study design render it difficult to draw solid conclusion about the efficacy of NMES.

Nutritional therapies that have emerged lately show that amino acid (AA) supplementation is beneficial for muscle health. A recent study has shown that taurine, a non-essential AA, has a therapeutic effect in the treatment of age-related sarcopenia. Taurine improves skeletal muscle regeneration, reduces chronic inflammation, and decreases high levels of oxidative stress thereby counteracting the effects of aging (Barbiera et al., 2022). Another recent study has shown that supplementation with essential AA reduces the loss in muscle volume in immobilized older adults recovering from total knee arthroplasty (Dreyer et al., 2018). The branched-chain AA leucine and its metabolite, β-hydroxy-β- methylbutyrate, activate mTORC1, increasing protein synthesis (Kamei et al., 2020). β-alanine levels are reduced in the skeletal muscles of aged mice, while β-alanine supplementation increases physical performance and improves endurance exercise-induced executive function in middle-aged individuals (Kamei et al., 2020). In elderly subjects with sarcopenia, an 18-month supplementation with a mixture of AAs significantly increases lean body mass associated with a significant decrease in circulating TNF levels and a marked increase in IGF-1 levels (Solerte et al., 2008). Preclinical evidence has shown that, in addition to AA, various nutrients including fatty acids (omega-3 and omega-6) and vitamins (C, D, E) are involved in modulating muscle protein synthesis and degradation and mitochondrial metabolism and energy production, thus improving muscle mass, strength, and function (Lee et al., 2022). Currently, the standard treatment approaches for sarcopenia are resistance exercise, protein supplementation, and vitamin D administration (Morley, 2016).

### Targeting inflammatory pathways

As inflammation is an important biological process involved in muscle wasting, especially in cancer-associated cachexia, several preclinical studies on rodent cancer models have reported that inhibiting IL-6 and its signaling pathways may have beneficial effect on muscle wasting (Belizário et al., 2016). The earliest studies over 20 years ago have shown that blocking the IL-6 receptor prevents muscle atrophy in a tumor-bearing mouse model *via* the modulation of the lysosomal and ATP-ubiquitin-dependent proteolytic pathways (Fujita et al., 1996). The study conducted in mice bearing human tumors shows that tumor cell-secreted IL-6 directly contributes to body weight loss while the administration of a human-mouse chimeric monoclonal antibody reverses muscle wasting, highlighting the potential role for IL-6 as pro-cachectic agent and the potential of its neutralization for treating cachexia (Zaki et al., 2004). In the same vein, targeting an IL-6 downstream signaling pathway has been shown to have a beneficial effect on protecting muscle against cachexia (Belizário et al., 2016). Bonetto *et al.* (2012) have shown that the inhibition of the STAT3/JAK pathway, downstream from IL-6 signaling, reduces cancer-induced cachexia and may be an interesting therapeutic target (Bonetto et al., 2012). Furthermore, in a model of denervation-induced skeletal muscle atrophy, blocking IL-6 with tocilizumab or the pharmacological/genetic inhibition of the JAK/STAT3 pathway in skeletal muscle both suppress muscle atrophy and inhibit mitophagy, leading to a decrease in the expression of atrophic genes (*MuRF1* and *MAFbx*) and autophagy-related genes (*PINK1*, *BNIP3*, *Beclin 1*, *ATG7*, and *LC3B*) (Huang et al., 2020). Targeting IL-6 or its downstream mediators is thus a promising therapeutic approach for preventing muscle atrophy.

Targeting the NF-κB pathway and the UPS system are also of interest in preventing muscle atrophy as their dysregulation is associated with different pathologies that cause muscle atrophy. Preclinical studies using MG132, a specific reversible proteasome inhibitor, have shown that MG132 blocks the degradation of ubiquitin-conjugated IκB, thereby inhibiting NF-κB activation and reducing cachexia (Inoue et al., 2009) (Zhang et al., 2013). The RANKL/RANK interaction activates NF-κB and we have previously shown, in dystrophic mice, that an anti-RANKL treatment improves muscle function and integrity and increases the proportion of anti-inflammatory and non-cytotoxic M2 macrophages (Hamoudi et al., 2019). In postmenopausal women, neutralizing RANKL using a monoclonal antibody (denosumab) treatment for more than 3 years improves appendicular lean mass and handgrip strength compared with an untreated group (Bonnet et al., 2019). In a mouse model of non-metastatic ovarian cancer cachexia, where elevated RANKL levels cause muscle atrophy, the administration of anti-RANKL has been shown to reduce muscle mass loss and dysfunction (Pin et al., 2022). Interestingly, full-length OPG, which contains four RANKL-binding domains, two TRAIL-binding domains, and one heparin-binding domain has been shown to be effective in preserving muscle function and integrity in dystrophic mice and is even superior to anti-RANKL, suggesting that full length OPG also acts *via* a mechanism that is independent of RANKL (Dufresne et al., 2018). In dystrophic mice, OPG improves muscle function, attenuates muscle inflammation, and improves muscle integrity by modulating Ca^2+^ homeostasis (Dufresne et al., 2015) (Dufresne et al., 2018). Alternative approaches targeting NF-κB/TNF-α or UPS could thus reduce muscle atrophy (Fang et al., 2021).

Megestrol acetate (MA) is currently the only drug used to fight both cachexia by stimulating appetite and certain cancers by acting as an antineoplastic agent (Wen et al., 2012). The combination of MA and thalidomide, which is known to have immunosuppressive effects, is superior to MA alone and improves the condition of patients with cachexia by reducing TNF-α levels (Wen et al., 2012). Lastly, targeting oxidative stress in the elderly appears be another potential therapeutic strategy. Allopurinol is in the xanthine oxidase inhibitor family of medications and reduces oxidative stress. A retrospective study in elderly subjects taking allopurinol as a medication has shown that they exhibit a greater degree of improvement in muscle function during rehabilitation, highlighting the potential beneficial effect of allopurinol on skeletal muscle (Beveridge et al., 2013). As inflammatory pathways are directly involved in inducing protein breakdown, targeting these pathways is a major focus of developing new therapeutic approaches to fight muscle atrophy.

### Targeting protein synthesis and degradation

Therapies targeting endogenous regulators of muscle atrophy such as TGF-β-like molecules, including the myostatin/TGF-β/activin pathway, have been tested preclinically given their interaction with activin receptor complex (ActRIIB), which induces the activation of SMAD2/3, which in turn causes the inhibition of Akt and results in a decrease in protein synthesis and cell differentiation (Morissette et al., 2009) (Trendelenburg et al., 2009). Different strategies have been developed to target endogenous regulators of muscle atrophy, including the neutralization of myostatin using myostatin antibody, the memetic endogenous neutralization of myostatin by follistatin, or the blockage of ActRIIB (Smith and Lin, 2013). However, clinical results have been very disappointing as they are not very efficacious and have significant adverse effects (Sartori et al., 2021) (Golan et al., 2018). Nonetheless, a novel strategy for inhibiting myostatin using a monoclonal antibody that selectively binds to the autoinhibited precursor form of myostatin and prevents its maturation has shown promising results in animals, with a reduction in muscle atrophy and an improvement in muscle strength and function (Pirruccello-Straub et al., 2018). In cachexia-associated muscle atrophy, the pharmacological blockade of myostatin/activin has been found to be the most effective therapy (Estelle et al., 2014). The use of β-blockers may be a potential strategy for treating cachexia given that the pathology is associated with weight loss and that β-blockers have effects on survival and weight gain by inhibiting catecholamine-induced lipolysis and decreasing insulin sensitivity (Kung et al., 2010). The β-blocker carvedilol has been shown to attenuate and partially reverse cachexia in patients with chronic HF (Clark et al., 2017). For GC-induced skeletal muscle atrophy, an emerging approach targeting urotensin-II (U-II) has shown interesting results as a novel therapeutic strategy. As GC increases the expression of U-II, antagonizing urotensin signaling improves muscle atrophy by increasing PI3K/Akt/mTOR levels and inhibiting UPS (Yin et al., 2022). Lastly, the use of anabolic hormones as therapeutic agents to treat muscle atrophy has attracted interest but often the effects have been associated with significant adverse effects (Morley, 2016). For example, the administration of high doses of testosterone has been shown to improve muscle mass and function but has several potentially limiting side effects (Morley, 2016). The same applies to ghrelin as its adverse effects need to be taken into consideration given that it increases IGF-1 levels, which may induce diabetes or insulin resistance (Yin et al., 2021). IGF-1 has shown very interesting effects on various neuromuscular diseases in preclinical studies. However, clinical trials have failed to reproduce these results (Song et al., 2013). Given the lack of effective treatments, major research efforts are ongoing to develop new therapeutic strategies that target muscle atrophy. The combination of exercise, nutraceutical, and pharmacological approaches that target multiple pathways may be required to effectively reduce muscle loss in aging and disease conditions.

## Conclusion

Muscle wasting and atrophy occur under different physiological and pathological conditions and involve a variety of cellular and molecular changes that may differ depending on the situation and the muscle fiber phenotype. Muscle atrophy in microgravity and the HS model primarily affects postural and slow-twitch muscle fibers. The mechanisms involved are complex and are not yet fully understood. Age-related sarcopenia is a morbidity that causes many complications and that is often associated with other co-morbidities such as osteoporosis, diabetes, and HF. Each of these morbidities causes muscle atrophy by modulating protein synthesis and degradation pathways and hormonal signaling, and by impairing mitochondrial and lysosomal functions. Pathological situations such as cancer cachexia involve an impairment of the protein degradation pathway, the modulation of inflammation, and apoptosis, whereas, in muscular dystrophies, the rise in cytosolic Ca^2+^ levels, muscle damage, and chronic inflammation are mainly responsible for muscle atrophy and wasting. While we have reviewed the mechanisms involved in the atrophy of skeletal muscle, we have also referenced the double-edged sword of inflammation in muscle repair. Although much is known about muscle wasting, it remains a focus of much ongoing research, and while many therapeutic approaches have been investigated in numerous preclinical studies, the clinical therapeutic armamentarium for treating muscle disorders, especially muscle wasting, has to be expanded, hence the need for more investigations.
